# Bryonia—Some Notes

**Published:** 1889-06

**Authors:** H. E. Potter

**Affiliations:** Clifton, Kansas


### BRYONIA—SOME NOTES—SOME MISTAKES.

Last October was called to see Harry S., æt. six years. Found he had been taking Acon. and Phos. alternately every hour for two or three days—on general principles, I suppose.

His malady seemed to be of a periodic character; there were fever (102°), intense thirst, excessive irritability, and a desire to lie perfectly quiet on the painful side (right). The least motion and even the respiratory effort greatly aggravated the pain. He was constipated, only meagre burnt stools had been secured for several days by assiduous repetition of enemata.

I diagnosed a Bry. case and prescribed accordingly; instructing the mother to discontinue the medicine as soon as improvement was apparent. The following day I called, finding my patient much improved. The medicine had been stopped after the second or third dose, given at two-hour intervals. The bowels moved naturally without any injection.

Case No. 2.—Mr. S., a merchant, who had no use for little pills, got at outs with the local allopathic brethren, and sent for me to give him a hypodermic of Morphine. I don't own a hypodermic syringe.

The man had been suffering for several days with influenza, which had become complicated with pleurodynia. Of course, he was in that condition when relief from suffering was very essential. He had for some time been taking Aconite and Bry. alternately every hour, which remedies had been prescribed by a homœopathic (?) druggist. Well, I'm not a "faith healer," so didn't try to fathom his mental evolvements as I told him 'twas not Morphine, but a homœopathic remedy which he needed. He said he didn't care a —— what did it so the pain was taken out of his side, that he might cough without killing him. I studied the case closely, and finally prepared some Bry. He took it, remarking, "Same old gag," and doubtless thinking he'd be obliged to get some Morphine on his own account at last; but two days later he was attending to his business, which he had not done for a week. It is only fair to conclude, as he very sensibly did, that the *one* remedy cured him whereas *two* had failed.

Case No. 3.—Mrs. B., æt. thirty-five years, recently passed through her sixth confinement. While making my first visit after the advent of the baby she said she never had had other than a "colicky baby," and that she was always constipated at such times. This constipation seemed to point to Bry., which was given in form of No. 30 pellets, six pellets to be taken three times daily when necessary, but to discontinue them as soon as relieved.

Some improvement was noticed after having taken them the first day; but I suppose she thought if a little was good more would be better. At any rate, she continued to take the pellets till she had a characteristic Bry. chill and many other pathogenetic features of the drug, among which were insatiable thirst, diarrhœa, and a desire to be perfectly quiet. Had I not feared disastrous results to the babe, I would have encouraged her to continue the proving; but she soon recovered on discontinuing the medicine, and now does not take a second dose of any medicine except as ordered. I do not know the exact potency of the remedy used in those three cases; I began with the 3x some two years ago and have been filling up since, till I suppose it now to be between the tenth and thirtieth. H. E. POTTER, M. D.

CLIFTON, KANSAS.

